# Towards Intelligent Interpretation of Low Strain Pile Integrity Testing Results Using Machine Learning Techniques

**DOI:** 10.3390/s17112443

**Published:** 2017-10-25

**Authors:** De-Mi Cui, Weizhong Yan, Xiao-Quan Wang, Lie-Min Lu

**Affiliations:** 1 Anhui and Huaihe River Institute of Hydraulic Research, No. 771 Zhihuai Road, Bengbu 233000, China; cdm@ahwrri.org.cn (D.-M.C.); wxq@ahwrri.org.cn (X.-Q.W.); llm@ahwrri.org.cn (L.-M.L.); 2 GE Global Research Center, Niskayuna, New York, NY 12309, USA

**Keywords:** deep foundation, defect detection, extreme learning machine, neural network, non-destructive evaluation, pile integrity testing, wavelet decomposition

## Abstract

Low strain pile integrity testing (LSPIT), due to its simplicity and low cost, is one of the most popular NDE methods used in pile foundation construction. While performing LSPIT in the field is generally quite simple and quick, determining the integrity of the test piles by analyzing and interpreting the test signals (reflectograms) is still a manual process performed by experienced experts only. For foundation construction sites where the number of piles to be tested is large, it may take days before the expert can complete interpreting all of the piles and delivering the integrity assessment report. Techniques that can automate test signal interpretation, thus shortening the LSPIT’s turnaround time, are of great business value and are in great need. Motivated by this need, in this paper, we develop a computer-aided reflectogram interpretation (CARI) methodology that can interpret a large number of LSPIT signals quickly and consistently. The methodology, built on advanced signal processing and machine learning technologies, can be used to assist the experts in performing both qualitative and quantitative interpretation of LSPIT signals. Specifically, the methodology can ease experts’ interpretation burden by screening all test piles quickly and identifying a small number of suspected piles for experts to perform manual, in-depth interpretation. We demonstrate the methodology’s effectiveness using the LSPIT signals collected from a number of real-world pile construction sites. The proposed methodology can potentially enhance LSPIT and make it even more efficient and effective in quality control of deep foundation construction.

## 1. Introduction

Assessing the structural integrity of deep foundation elements such as drilled or driven piles has always been a critical quality control task in the construction industry. Over the years, many nondestructive evaluation (NDE) methods have been developed for reliably assessing the integrity of piles, for example, low strain pile integrity testing (LSPIT), high strain pile integrity testing (HSPIT), cross-hole sonic logging (CSL), single hole sonic logging (SSL), and gamma-gamma density logging (GDL) [1]. Among these different integrity testing methods, LSPIT, also called the sonic echo test, is probably the most popular one widely used in various parts of the world. The popularity of LSPIT comes from the fact that it is effective in detecting major discontinuities or defects, such as cavities, cracks, necking, bulging, and soil inclusions, and relatively simple to perform in the field [2].

LSPIT works by following one-dimensional wave propagation theory [3]. A stress wave introduced by the blow of a hand-held hammer on the pile top propagates axially along the pile, and reflections are generated whenever the stress wave encounters impedance changes (discontinuities). Theses reflections are measured with the acceleration transducer installed on pile top. These reflections are later analyzed based on one dimensional stress wave analysis.

An entire LSPIT involves two parts: (1) field testing—signal acquisition and (2) signal interpretation—qualitatively and quantitatively assessing pile integrity by interpreting the signals (velocity reflectograms) collected from the field test. While field testing is relatively simple and can be performed fairly quickly by qualified personnel, assessing the pile integrity by interpreting the test signals is still quite challenging and involving. That is because many factors affect the wave propagation and thus the reflection signals. In particular, interpreting the signals for detecting pile defects to determine defect types and locations requires experienced personnel with good knowledge of wave propagation theory, soil mechanics, and piling construction techniques, in addition to a good understanding of LSPIT itself. As a result, interpreting field-obtained signals is currently performed manually by experienced experts only. When the number of piles tested is large, the experts may be overwhelmed by the large amount of manual interpretation work, which leads to one of the two potential consequences: (1) the inability to complete the pile integrity assessment on time, resulting in delays of the foundation construction schedule, and (2) an increased possibility of errors (e.g., mis-detection of defect piles) due to lack of time for the expert to perform a thorough interpretation of test signals and in-depth analysis of soil conditions. Therefore, techniques that enable speedy and reliable interpretation of LSPIT signals, while minimizing experts’ effort at the same time, are of great business value.

There have been a few studies on intelligent interpretation of LSPIT signals in recent years. For example, in [4], a neural network classifier was used for detecting and identifying defects of concrete piles. However, their method worked based on both the known ideal reflectograms and the field test PIT signals, where the ideal reflectograms were numerically obtained through finite element method (FEM) and scaled boundary FEM (SBFEM). The requirement of ideal reflectograms limits its broad applications. Other works, e.g., [5], also used neural network and worked on numerically simulated reflectograms.

In this paper we proposed a computer-aided reflectogram interpretation (CARI) methodology that interprets field-generated LSPIT signals/reflectograms directly without a numerical model of the piles. Our proposed CARI methodology is based on advanced signal processing and machine learning techniques to analyze and interpret PIT signals more effectively and efficiently. Specifically, wavelet analysis is used to extract important features from the raw LSPIT signals, and such extracted features are then used as input to extreme learning machines (ELM) [6], an advanced artificial intelligence technique, for pile defect detection. Since many factors affect the reflectograms, fully automatic interpretation without human intervention is practically infeasible. That is exactly why ASTM D5882 specifically requires “Engineers with specialized experience in this field are to make final integrity evaluation” [7]. Realizing this, we deliberately develop our CARI methodology such that it is not completely free of human experts’ involvement in interpretation, but rather it is designed to greatly reducing human experts’ effort in interpretation. Specifically, the proposed methodology eases experts’ interpretation burden by quickly screening all tested piles and identifying a small number of suspected piles for experts to do manual interpretation. Note that, in a typical real-world pile foundation construction, the number of defect piles is normally small compared to the number of normal piles.

ELM, a new family of neural networks, has been actively studied in the past a few years [6]. The applications of ELM cover diverse domains, including image analysis [8,9], medical science [10], and text analysis [11]. Recently, ELM has also been applied to fault detection and diagnosis of mechanical systems [12,13]. Using ELM as a means for intelligent interpretation of PIT signals has never been done, to the best of our knowledge. Thus, our contribution in this paper is primarily to introduce ELM-based CARI methodology for intelligent and fast interpretation of LSPIT signals. We also demonstrate the effectiveness of the proposed methodology using a large number of LSPIT signals collected from various real-world engineering construction sites. The proposed CARI methodology can potentially enhance LSPIT as a NDE tool and make it even more efficient and effective in quality control of deep foundation construction.

The remainder of our paper is organized as follows. In Section 2, we provide background information about LSPIT, including the principle of LSPIT and the prior work related to intelligent interpretation of LSPIT signals. Details of the proposed methodology are given in Section 3. Section 4 provides experimental results and discussion, while conclusions are given in Section 5.

## 2. Background 

### 2.1. Principle of LSPIT

**Figure 1:**
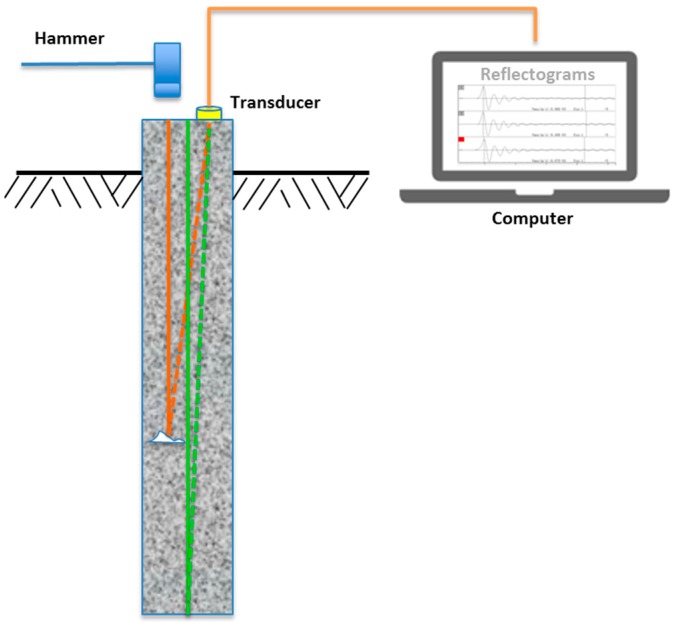
Schematic view of low strain pile integrity testing.

The low strain pile integrity test is an echo method for qualitative evaluation of the physical dimensions, continuity of a pile, and consistency of the pile material. The low strain integrity test has been used since the 1970s and has been standardized by ASTM D5882—Standard Test Method for Low Strain Impact Integrity Testing of Deep Foundations [7]. As specified in ASTM D5882, there are two testing methods for the low strain integrity test. One is the Pulse Echo Method (PEM), and another is the Transient Response Method (TRM). This study is concerned with the PEM where only pile head motion is measured and analyzed for pile integrity evaluation. As illustrated in Figure 1, to perform LSPIT with PEM, the pile head is taped with a hammer, which generates the stress wave (sound wave) that travels through the pile length and reflects back to the pile head. The acceleration transducer placed on top of pile head measures the response of the stress wave. The measured acceleration is integrated into a velocity signal, popularly called “velocity reflectogram” or simply “reflectogram,” which offers a great amount of information for both the qualitative and quantitative assessment of pile integrity.

The well-known wave equation in a one-dimensional elastic rod (ignoring the soil resistance) is given as (assuming pile material is homogeneous)

(1) $$\frac{\partial^{2}u\left(x,t\right)}{\partial t^{2}}-C^{2}\frac{\partial^{2}u\left(x,t\right)}{\partial x^{2}}$$

C=Eρ is the wave propagation velocity, where *E* and *ρ* are the dynamic Young’s modulus and the mass density of the pile material, respectively, and *u(x,t)* is the axial displacement of a mass point at section *x* and time *t*.

The impedance, defined as Z=EAC=AEρ, where *A* is the cross-sectional area of the pile, is a metric for measuring pile resistance change with respect to velocity. Any change in *A*, *E*, *ρ*, or a combination of them will result in an impedance change or discontinuity. When a wave traveling in a rod meets such a discontinuity, one part of it will be reflected back while another part will go on beyond the discontinuity. Assume at the discontinuity section the impedance changes from $Z_{1}$ to $Z_{2}$. Then, the amplitude of reflected wave, $V_{R}$, is related to the amplitude of the incoming wave, $V_{I}$, as follows:

(2) $$V_{R}=\frac{Z_{1}-Z_{2}}{Z_{1}+Z_{2}}V_{I}=\frac{\rho_{1}C_{1}A_{1}-\rho_{2}C_{2}A_{2}}{\rho_{1}C_{1}A_{1}+\rho_{2}C_{2}A_{2}}V_{I}$$

### 2.2. Related Work

As one of the most popular NDE methods in the pile foundation construction industry, LSPIT has been widely used for qualitative evaluation of the physical dimensions, continuity of a pile, and consistency of the pile material since 1970s. Over the years, research efforts on improving the LSPIT method have been focused on two separate directions: (1) theoretical analysis and understanding of LSPIT mechanism and (2) intelligent and automatic interpretation of test results. While the first direction is important and has attracted much research interests (e.g., [14]), our paper is concerned with the second direction, i.e., automatic interpretation of LSPIT signals. Toward intelligent interpretation of LSPIT signals, a limited number of studies have been on using advanced signal processing and artificial intelligence (pattern recognition) techniques for LSPIT signal analysis.

In his PhD thesis [15], Watson applied three different types of neural networks to LSPIT for aiding test signal interpretation. However, the data he used for training the neural network models was generated from the FEM models, rather than from field test signals. Others have also applied neural networks to PIT signals. For example, Zhang & Zhang [16] proposed using two ANN models for processing the PIT signals for diagnosing pile integrity. They used the first ANN model for identifying defect patterns and the second ANN model for assessing severity of the defects. The inputs to the ANN models include time-domain signals, the pile length, the cross-sectional area, and the wave velocity. Tam et al. [5] investigated using PNN (probabilistic neural networks) for diagnosing prestressed concrete pile defects.

More recently, Protopapadakis et al. [4] applied a genetically optimized neural classifier to identify neck and bulk defects of concrete piles. Instead of directly using test signals as inputs to the neural classifier, they used the difference between the test signals and the ideal waveform where the ideal waveform was generated numerically using FEM and scaled boundary finite element method (SBFEM). They also used the island generic algorithm (GA) to optimize the neural network structure.

In [17], Garcia et al. proposed using recurrence plots, a technique from Chaos theory for analyzing and interpreting pile test signals. They converted 1D reflectograms into 2D RP images and then found different characteristics of the 2D RP images associated with different conditions (normal and different defects).

Wavelet analysis is an advanced signal processing technique [18]. Being able to “look” at the signals through both a temporal and scale lens simultaneously, wavelet analysis can handle noisy and non-stationary signals much better than traditional Fourier transformation. Zhang et al. [19] proposed using wavelet analysis and neural networks for pile defect diagnosis.

Machine learning techniques have been widely used for condition-based maintenance (CBM), structural health monitoring (SHM), and non-destructive evaluation (NDE). References [20,21,22,23] provide an in-depth overview of applications of machine learning techniques. Extreme learning machines (ELM), an advanced artificial intelligence technique, has been successfully used in various applications in recent years [8,9,10,11,24,25,26,27]. Using ELM as a means for intelligent interpretation of PIT signals, however, has never been done before, to the best of our knowledge. 

## 3. Proposed Methodology

**Figure 2:**
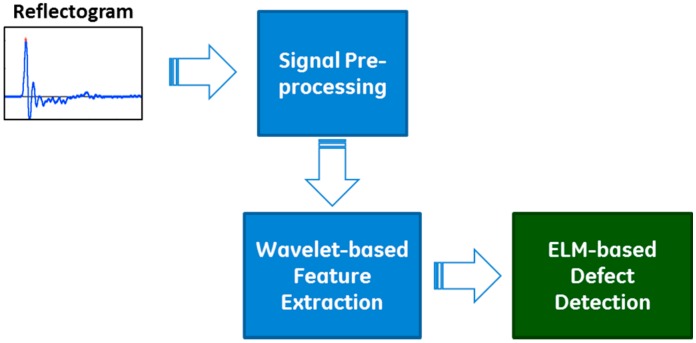
Overall structure of the proposed CARI methodology.

Aiming for automatic and intelligent interpretation of a large number of field test signals, we proposed the CARI methodology. The proposed methodology, built on the advanced signal processing and artificial intelligence techniques, consists of three components: (1) signal preprocessing, (2) Wavelet-based feature extraction, and (3) ELM-based defect detection, as shown in Figure 2. Detailed descriptions of these three components are given as follows.

### 3.1. Signal Preprocessing

To strengthen the reflectogram signal, thus for better interpretation of test results, several signal enhancement strategies are often required. First, the signals measured at the pile head need to be amplified exponentially for compensating stress wave signal weakening due to the shaft friction influence during wave traveling through the pile. Then, high frequency reflections exist in reflectograms due to the shear wave influence at the pile top and steel reinforcement inside the pile. Thus, low-pass filtering is also needed most of time to remove those high frequency reflections. We use wavelet decomposition to perform the filtering during the feature extraction process (See Section 3.2).

As will be discussed in Section 3.2, our feature extraction is wavelet decomposition–based, and feature calculations will be performed over the portion of reflectograms between pile top and toe reflections. Thus identification of reflections from reflectograms is needed for our proposed methodology. Reflections in reflectograms appear as waveform peaks. Reflection identification thus becomes a peak detection problem. In the literature, there are many peak detection algorithms. In this study, we use a simple peak detection algorithm that is based on the sign of first-order difference of the signal [28]. A peak occurs when the signal changes directions, that is, a peak is defined as the sign of signal differences changes from a streak of positives and zeros to negative. The pseudo code of our peak detection is shown in Algorithm 1.


**Algorithm 1.** Pseudo-code of our peak detection algorithm.**Inputs:**
$x=\left[x_{1},x_{2},\;\ldots ,\;x_{n}\right]$ // a n-point waveform            T              // A threshold value**Outputs:**
pK, pIdx // peaks and corresponding indices
1. Calculate the 1st-order difference of x, dx[i]=x[i]−x[i−1], i∈2, 3,…,n2. Determine the sign of dx[i], i∈2, 3,…,n        sdx[i]=+ 1 for dx[i]>0;sdx[i]=−1 for dx[i]<0;sdx[i]=0 for dx[i]=03. Search for the sign changes from +1 to −1        pIdx=∅;pK=∅    **for**
*j* = 2 to *n*          $\mathrm{if}\;\left(sdx\left[j\right]-sdx\left[j-1\right]=-2\right)\;\mathrm{then}\;pIdx=pIdx\cup^{\text{​}}j;pK=pK\cup^{\text{​}}x\left[j\right];\mathrm{end}$        **end**4. Remove those peaks with values being less than the threshold, T

### 3.2. Wavelet-Based Feature Extraction

Extracting a set of good features from the raw signals is almost always required in order to achieve better predictive models. In the domain of machine learning, feature extraction is regarded as the critical and labor-intensive task [29,30]. We have seen a wide range of feature extraction methods in literature, including the traditional statistical-based methods as well as the modern deep learning methods [31,32]. Given the fact that LSPIT signals (reflectograms) are highly non-stationary and noisy, in this paper, we propose using wavelet analysis to extract features from the reflectograms. Wavelet analysis is an advanced signal processing technique [18] and has been popularly used in various domains and applications [33,34,35,36,37,38,39,40].

**Figure 3:**
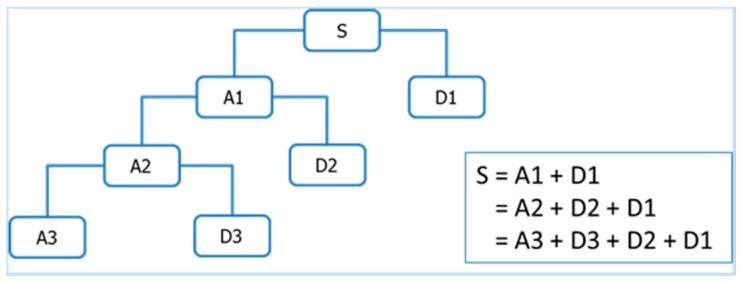
An illustration of 3-level wavelet decomposition.

Multi-resolution wavelet decomposition, a type of wavelet analysis, is to decomposes a signal into a bunch of orthonormal bases with different time and frequency resolutions [18]. As illustrated in Figure 3, for 3-level wavelet decomposition, the signal is represented by an approximation that contains the high-scale, low-frequency components of the signal and three details that represent the low-scale, high-frequency components of the signals. 

**Figure 4:**
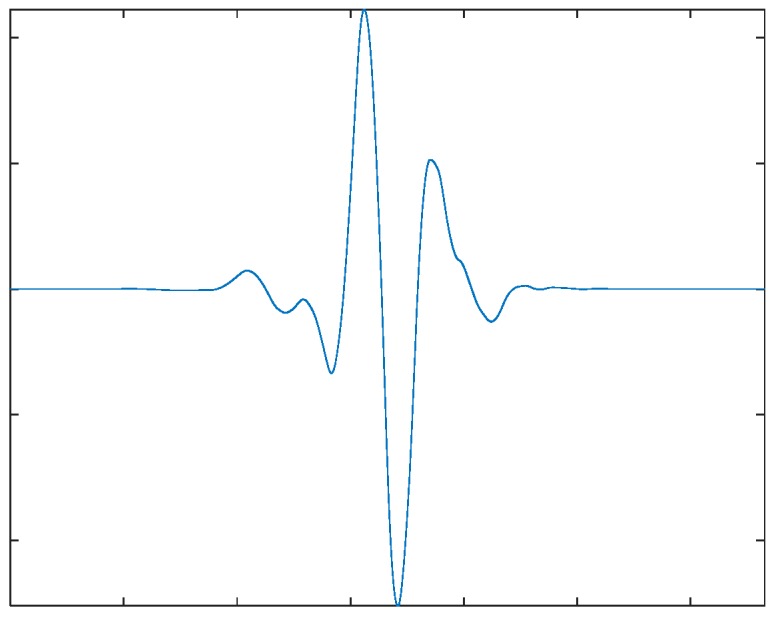
The seventh “symlet” wavelet function.

**Figure 5:**
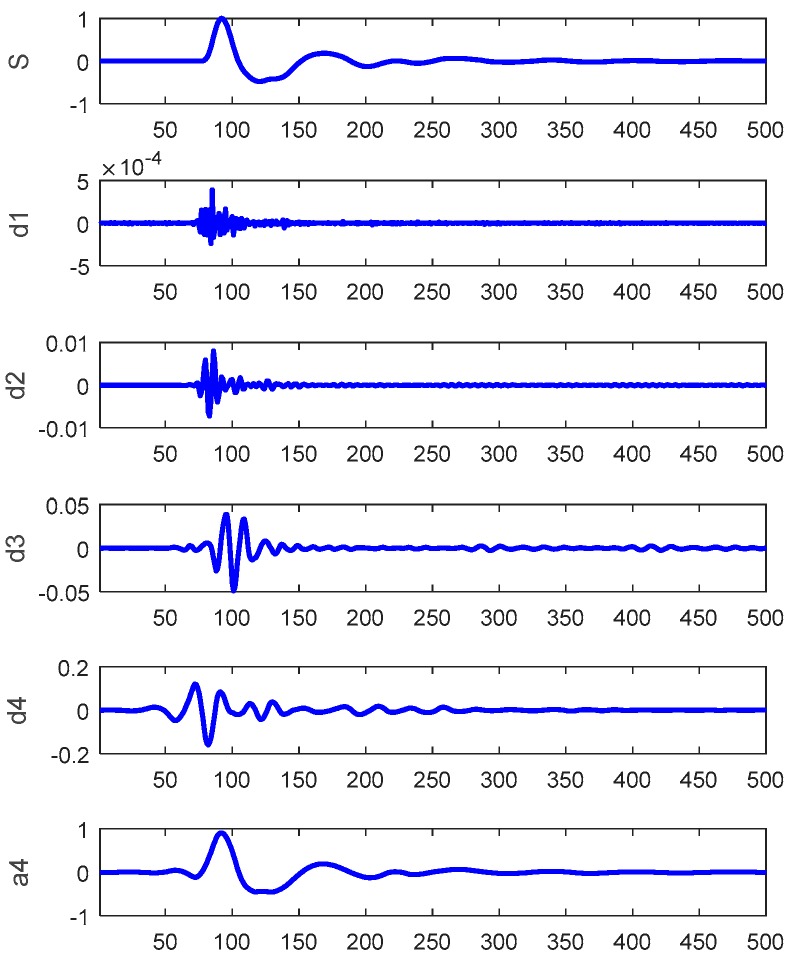
An illustration of 4-level wavelet decomposition of a reflectogram: s = original signal; d1 ~ d4 are the first through fourth details and a4 is the fourth approximation

For the LSPIT reflectograms concerned in this paper, we adopt 4-level wavelet decomposition to achieve a reasonable balance between the time and frequency resolutions. Based on visual analysis of reflectograms, we choose the seventh order “symlet” (see Figure 4 above) as the mother wavelet, which gives the wavelet shape that best matches the reflections of the reflectograms. Figure 5 shows an example of the reflectograms and the approximation and details resulted from the 4-level wavelet decompositions. We perform feature extraction on the fourth approximation (A4) and the fourth and thirrd details (D4 & D3) and ignore other higher frequency components. For each of the three selected bases, we extract seven features (defined below) from its wavelet coefficients, its reconstructed waveforms, and the spectrums of the reconstructed waveforms, respectively, which gives us a total of 21 features.

Let $x_{n},\;n=1,2,\ldots ,N$ be the time domain signals and $\left[p_{i},\;f_{i}\right],\;i=1,2,\ldots ,\;M$ be its corresponding spectrum, where $p_{i}$ and $f_{i}$ are the amplitude and the frequency at $i^{th}$ frequency bin, respectively. The seven features are defined as follows:

(1) Energy: $E=\sum_{i=1}^{N}x_{i}^{2}$; (2) Total power: $TP=\sum_{i=1}^{M}p_{i}$; (3) Mean power: MP=TPM; (4) first spectral moment (centroid): $M_{1}=\sum_{i=1}^{M}p_{i}f_{i}/TP$; (5) 2nd spectral moment (standard deviation):

(3) $$M_{2}=\sqrt{\sum_{i=1}^{M}{\left(f_{i}-M_{1}\right)}^{2}\cdot p_{i}/TP}.$$

(6) third spectral moment (skewness):

(4) M3=∑i=1M(fi−M1)3⋅piM23⋅TP

and (7) fourth spectral moment (kurtosis):

(5) M4=∑i=1M(fi−M1)4⋅piM24⋅TP

### 3.3. Defect Detection Using ELM

The CARI methodology proposed in this paper is for quickly assessing the pile integrity status based on the LSPIT signals collected from the field tests, that is, to determine whether or not the test pile has any defect, which is often called defect detection. Treating defect detection as a binary classification problem, we can then apply a classification method to solve it.

There are numerous classification methods available, including decision trees, various neural networks, different types of support vector machines, and ensemble learning based method, e.g., random forests and adBoost. In this study, we choose ELM as our defect detection (classification) model, simply because ELM has several unique properties that are well suited for our CARI methodology. The unique ELM properties are summarized as follows.

ELM, a new family of neural networks [6], involves a different way to determine the network parameters—the connection weights and biases between layers. More specifically, instead of learning all parameters as in conventional feed-forward neural networks, ELM’s connection weights and biases between the input and hidden layers are randomly generated and are kept fixed. ELM learning is then simply to determine the connection weights between the hidden and the output layers through solving a linear least squares problem [6]. Because of the special design of ELM, there is no need to go through an iterative optimization process for finding the optimal network parameters, as required in conventional neural networks. As the result, ELM is much faster than conventional neural networks with regard to learning. Also ELM seems to be more effective in handling problems with sparse data, i.e., small number of training samples. More importantly, ELM can achieve better performance than other machine learning methods (including SVM), based on several of recent studies (both empirical and analytical) [41,42].

For completeness in the paper, we briefly describe the essence of ELMs as follows. Please refer to [6,41,42] for more thorough discussion of ELMs.

Consider a set of *M* training samples, $\left(x_{i},y_{i}\right),\;x_{i}\in {\mathbb{R}}^{d},y_{i}\in {\mathbb{R}}^{k}$, where *d* and *k* are the dimensions of input and output spaces, respectively. The output of a single layer ELM network with *L* hidden neurons for an input vector, ***x***, can be expressed as

(6) $$f\left(x\right)=\overset{L}{\underset{i=1}{\sum }}\beta_{i}h_{i}\left(x\right)=h\left(x\right)\beta$$

In the above equation, $h_{i}\left(x\right)=G\left(w_{i},b_{i},x\right)$ ($w_{i}$ and $b_{i}$ are the randomly generated weights and bias) is the $i^{th}$ hidden neuron output of the input ***x***; G(w,b,x) is the activation function, which can be any nonlinear piecewise continuous function that satisfies the ELM universal approximation capability theorems [41]; β is the weight matrix that contains the weights connecting the hidden and output layers. $h\left(x\right)=\left[h_{1}\left(x\right),\ldots ,h_{L}\left(x\right)\right]$ is often called the L-dimension random feature space, or the ELM feature space.

ELM learning is simply to find the optimal β through optimizing the objective function as defined [42]:

(7) $$\mathrm{Minimize}:\;L_{p}=\frac{1}{2}{∥\beta ∥}^{2}+\frac{1}{2}C\sum_{i=1}^{N}{∥\xi_{i}∥}^{2}$$

$$\mathrm{Subject}\;\mathrm{to}:\;h\left(x_{i}\right)\beta =y_{i}^{T}-\xi_{i}^{T},i=1,\;\ldots ,\;N$$

where $\xi_{i}={\left[\xi_{i,1},\;\ldots ,\;\xi_{i,k}\right]}^{T}$ is the error vector of the training sample $x_{i}$, and *C* is a constant regularizing the ELM network complexity and its prediction performance.

The equivalent dual optimization objective function is

(8) $$L_{d}=\frac{1}{2}{∥\beta ∥}^{2}+\frac{1}{2}C\sum_{i=1}^{N}{∥\xi_{i}∥}^{2}-\sum_{i=1}^{N}\sum_{j=1}^{k}\alpha_{i,j}\left(h\left(x_{i}\right)\beta_{j}-y_{i,j}+\xi_{i,j}\right).$$

Solving the above optimization utilizing the Karush–Kuhn–Tucker (KKT) condition, we obtain the ELM’s input-output relationship function f(x) as follows [42]:

(9) $$f\left(x\right)=h\left(x\right)\beta =h\left(x\right)H^{T}{\left(\frac{I}{C}+HH^{T}\right)}^{-1}Y,\;\mathrm{when}\;N\;\mathrm{is}\;\mathrm{not}\;\mathrm{too}\;\mathrm{big}$$

(10) $$\mathrm{and}\;f\left(x\right)=h\left(x\right)\beta =h\left(x\right){\left(\frac{I}{C}+H^{T}H\right)}^{-1}H^{T}Y,\;\mathrm{when}\;N\gg L$$

where ***H*** is the hidden layer output matrix.

(11) $$H=\left[\begin{array}{c} h\left(x_{1}\right)\\ \vdots \\ h\left(x_{N}\right) \end{array}\right]=\left[\begin{array}{ccc} h_{1}\left(x_{1}\right) & \ldots & h_{L}\left(x_{1}\right)\\ \vdots & \vdots & \vdots \\ h_{1}\left(x_{N}\right) & \ldots & h_{L}\left(x_{N}\right) \end{array}\right]$$

To handle the situation where data has imbalanced class distribution, weighted ELM (WELM) has been proposed [43]. Let W be a *N* × *N* diagonal matrix and $W_{ii}=1/\#\left(C_{i}\right)$, where $\#\left(C_{i}\right)$ is the number of samples in the class that $i^{th}$ sample belonging to. With the W defined, the ELM output function (Equation (8)) becomes $f\left(x\right)=h\left(x\right)\beta =h\left(x\right){\left(\frac{I}{C}+H^{T}WH\right)}^{-1}H^{T}WY$.

## 4. Experimental Results and Discussion

### 4.1. The Reflectogram Data

To demonstrate the capability of the proposed CARI methodology for identifying defect piles based on LSPIT signals, in this section we apply the proposed CARI to a large number of reflectograms collected from various real-world foundation construction sites. Table 1 summarizes the piles considered for our experiments. A total of 923 piles from 27 construction sites are considered. All the piles are friction piles and have four different types: Type I—pre-cast RC pipe pile, Type II—prestressed high strength concrete driven pipe pile, Type III—RC cast-in-place (bored) pile, and Type IV—RC cast-in-place (dogged) pile. Pile lengths vary from 5 m to 18.8 m (see Table 1). For all of the piles considered, the LSPIT tests were performed by experienced experts from our institute—Anhui and Huaihe River Institute of Hydraulic Research (AHRIHR). Manual interpretation of the test signals (reflectograms) were also performed by the experts. The detailed test reports for each of the construction sites and associated test signals have been well documented and archived in the institute’s database.

**Figure 6:**
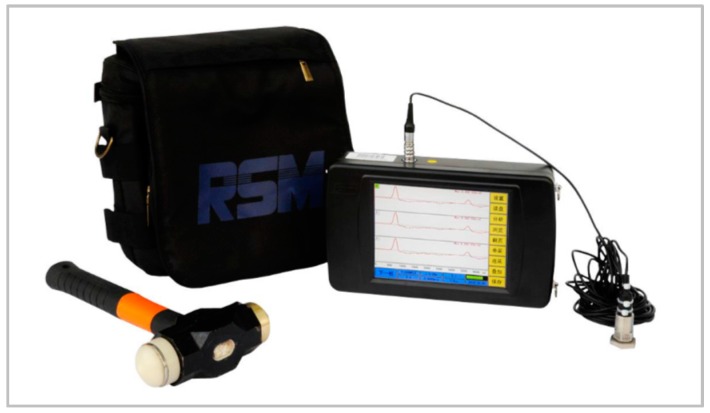
Our LSPIT equipment.

Field LSPIT testing was performed using the RSM-PRT Low Strain Pile Integrity Tester manufactured by Wuhan Sinorock Technology Co., Ltd., Hubei, China. Figure 6 shows the test equipment.

The sampling rate is 50 kHz and the total number of samples per pile is 1024. During field testing, for each pile three reflectograms were obtained by tapping three different spots of the pile top with different hammers with different weights. Only one reflectogram containing clear pile features was chosen for processing and storing in the database.

Since all defective piles have been verified by the experts and/or in the field, we can assume they are truly defective, thus our ground truth for defect detection model evaluation. We will evaluate our proposed CARI by comparing the classification results of CARI against the ground truths (normal and defective piles) described in the test report.

As shown in Table 1, in total we have 860 normal piles and 63 defective piles. Our goal here is to design a binary classifier to distinguish defective piles from normal piles. Since the number of defective piles is much smaller than the number of normal piles, we have so-called “imbalanced data distribution” issue, an important machine learning issue that requires special attention in classifier building. In this work we address the data imbalance issue by using the weighted ELM described in Section 3.

### 4.2. Detection Performance Evaluation and Methods

To assess the performance of anomaly detection models, we use the Receiver Operating Characteristic (ROC) curves and the related area-under-curve (AUC), the well-known performance metrics, as the classification performance measures for performance comparison. ROC curves represent the tradeoffs between true positive rate (TPR) and positive rate (FPR) [44,45]. ROC curves are good for visual comparison of classifier performance. To quantitatively assess classifier performance, the area under the curve (AUC) calculated from the ROC curve is often used. The AUC is not sensitive to the class sample distribution and represents the classification performance at various decision thresholds.

In terms of actual model evaluation method, we use five-fold cross-validation. In fact, k-fold cross-validation is a well-known model evaluation method that has been popularly used in many predictive modeling applications [46]. To ensure a robust comparison we run the five-fold cross-validation 10 times, each time with different randomly splitting of the five folds of the data. 

### 4.3. Results and Discussion

For ELM classifier design, the number of hidden neurons is fixed to 500, as suggested in [6]. The activation function for the hidden neurons is the sigmoid function, $G\left(w,b,x\right)=1/(1+\exp\left(-\left(w^{T}x+b\right)\right)$. The model parameter, C, is empirically determined via cross-validation by trying 20 different values, i.e., $C=\left[2^{-9},2^{-8},\ldots 2^{10}\right]$. For comparison purpose, we also implement a conventional feed-forward neural network (FFNN) as the classification model, using the same data, the same extracted features and the same evaluation method. For the FFNN design we also use the sigmoid function as the activation function, and the number of hidden neurons varies from 5 to 50 with an increment of 5.

**Figure 7:**
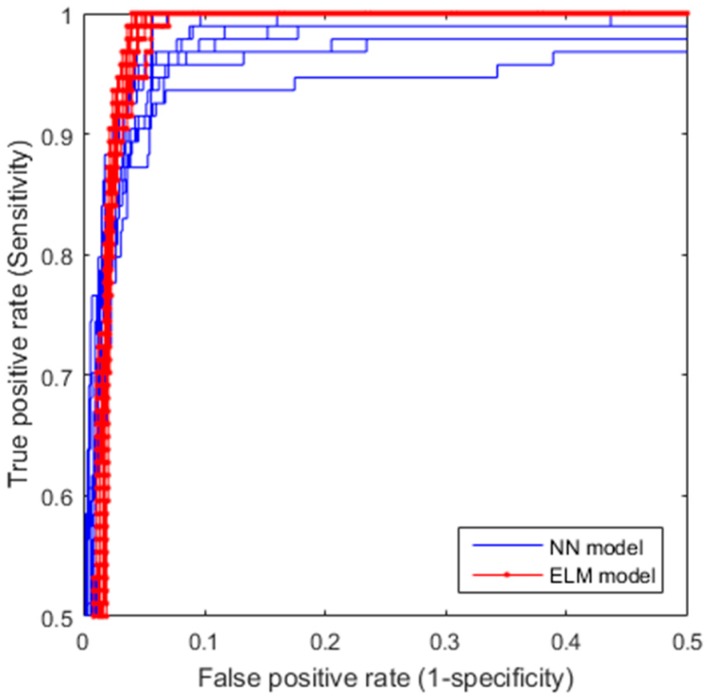
Receiver Operating Characteristic (ROC) curves for extreme learning machines (ELM) and feed-forward neural network (FFNN) models.

Figure 7 shows the ROCs of the 10 5-fold cross-validation runs for both ELM and FFNN models, respectively, where y-axis (sensitivity) is the TPR and x-axis (1—specificity) is the FPR. From Figure 7, one can visually see that the ELM model not only has better classification performance (higher ROC curves), but also is more robust (smaller variation for different runs) than the FFNN does. To perform a quantitative comparison of the ROCs, area-under-curve (AUC) for each of the ROCs is calculated. Table 2 shows the means and the standard deviations of AUCs of the 10 random runs for the two models compared, which confirms our visual observation, that is, ELM outperforms FFNN in terms of classification accuracy (larger mean value) and robustness (smaller variance).

For reflectogram interpretation concerned in this paper, since our goal is to identify a small number of suspected piles from a large number of the tested piles in order to reduce the expert’s effort on analysis and interpretation, we would like our CARI methodology to have the highest sensitivity (TPR) as possible to minimize misdetection, and our false positive rate, equivalent to how many piles the human expert needs to interpret, to be as small as possible. If we set our true positive rate to be 100%, i.e., no misdetection at all, the average false positive rate obtained from the ROC curves is 5.55% as shown in the confusion matrix (Table 3).

To help better understand the value of the proposed CARI methodology, let use a hypothetic example. Assume a construction site has a total of 101 piles (1 defective and 100 normal piles). Without using the proposed CARI, the human expert has to examine LSPIT signals of all 101 piles in order to identify the one defective piles. Now with the proposed CARI, he or she only needs to interpret signals of seven piles: one defective pile and the 6 (100 × 5.55% ≅ 6) false identified piles. That is, the proposed CARI methodology reduces human experts’ interpretation effort from 101 piles to seven piles, while ensuring the one defective pile is correctly identified. 

The results shown above are the cross-validation outcomes without discerning different pile types, which represent an overall classification performance. These results also give a good indication on how well the proposed CARI generalizes across different construction sites. To assess how well our proposed methodology generalizes cross different pile types, we conduct the pile type-wise cross-validation. That is, for the dataset concerned in this paper, which has for pile types (see Table 1), we perform 4-time type-wise cross-validation. Specifically, each time we leave out all samples associated with one pile type and train the ELM detection model with all remaining samples; and then test the model on the samples of the leave-out type. Table 4 shows the type-wise cross-validation results.

Comparing Table 3 and Table 4, one can see that our model classification performance (true detection rate and false positive rate) degrades under the type-wise cross-validation, indicating that reflectogram characteristics among different pile types are significantly different. Essentially, the type-wise cross-validation assesses how well our detection model performs on new, unseen pile types that have different reflectogram characteristics. We argue that making our detection model generalize well to a new pile type with different characteristics is practically unnecessary in real-world applications. Given the fact that different pile types have different reflectogram characteristics, in order to apply our methodology to a new pile type, we just need to update our detection model whenever samples (i.e., reflectograms of LSPIT tests) of the new pile type are available. An alternative solution would be to simply build a specific detection model for each of the pile types. Hence, the less effectiveness of our methodology in generalizing to new pile types should not hinder the applicability of our methodology in real-world applications.

## 5. Conclusions

Low strain pile integrity test (LSPIT) is a mature method and has been popularly used worldwide for assessing pile integrity. Test results interpretation, an important task of LSPIT, is currently still a manual process performed by experienced experts. Such a manual process is labor-demanding and also becomes a great burden in situations when field testing and the pile integrity assessment results need to be completed on a tight schedule. Technologies that can enhance LSPIT by speeding up the interpretation process, thus being able to have a quick turnaround in completing the test, are of great business value and are in great need. Our study in this work is an effort toward addressing this need. Realizing that fully automated interpretation is practically infeasible since many factors affect the reflectograms, in this study, we propose the CARI methodology that does not completely free human experts’ involvement in interpretation, but rather greatly reduces human experts’ effort in interpretation. Since human experts only need to look at a few suspected piles identified by our CARI methodology, they can afford to perform more thorough analysis by considering soil condition and pile construction information, thus obtaining more reliable assessment results. As the result, we would expect the integrity assessment obtained would be more reliable and more accurate, enabling LSPIT to be more efficient and more effective.

Using a reasonable number of reflectograms collected from real-world piles in various foundation construction sites, we have demonstrated that the proposed CARI methodology is effective in detecting defective piles while maintaining the false positive rate reasonably low. We also noted that different pile types have different reflectogram characteristics, and thus cautious measures are required when applying the methodology to a new, unseen pile type.

In future, we will continue to validate the proposed methodology using more real-world piles with more diverse types and soil conditions. We will also explore other different modeling techniques, for example, ensemble of ELM models, to further improve the classification performance. It is also our interest to expand the capabilities of the proposed methodology to cover defect identification, i.e., identifying defect types of piles.

## Figures and Tables

**Table 1:** Pile and construction site summary.

No	Site Name	# of Piles	# of Defect Piles	Pile Length (m)	Pile Type
**1**	Jing-Ao Bldg.#7	21	0	11	I
**2**	Jing-Ao Bldg.#9	19	0	14	I
**3**	Jing-Ao Bldg.#10	20	0	12	II
**4**	Jing-Ao Bldg.#26	15	0	11	II
**5**	Jing-Ao Bldg.#28	20	0	12	II
**6**	Jing-Ao Bldg.#29	38	0	13	II
**7**	Jing-Ao Bldg.#30	19	0	13	II
**8**	Jing-Ao Bldg.#31	20	0	13	II
**9**	Jing-Ao Bldg.#32	37	0	14	II
**10**	Jing-Ao Bldg.#35	36	0	12	II
**11**	Jing-Ao Bldg.#36	36	0	12	II
**12**	Jing-Ao Bldg.#37	20	0	13	II
**13**	Jing-Ao Bldg.#38	35	0	12	II
**14**	Ye-Ji 35kvRoad	66	8	6.8–10.5	III
**15**	Fong-Fang RailroadBldg #4	46	3	11, 12, 14	III
**16**	Fong-Fang RailroadBldg #5	47	2	11, 12, 14	III
**17**	Fong-Fang RailroadBldg #7	50	6	11, 12, 14	III
**18**	Fong-Fang Railroad Pump Station	34	3	16, 18	III
**19**	Shang-Shui-Guang	37	6	10.5	III
**20**	Yi-Shi-Jia Bldg # 3	27	4	16, 17	II
**21**	Yi-Shi-Jia Package Bldg	33	3	15, 16	II
**22**	Yi-Shi-Jia Bldg # 2	56	3	17	II
**23**	Ying-Chao-Yang	6	6	9.8–18.8	II
**24**	Yi-Shi-Jia Bldg # 1	65	3	16, 17	III
**25**	Lu-An FongHuanBldg # 5	87	7	5–9.47	IV
**26**	Long-Hua 35KV Engr. Site	19	8	7.5–13.5	IV
**27**	Bing-He Shuandung Power Station	14	1	9–12	IV
	Total	923	63	N/A	N/A

**Table 2:** Areas-under-curve (AUCs).

	AUCs
**ELM**	0.9841 ± 0.0022
**FFNN**	0.9780 ± 0.0112

**Table 3:** Confusion matrix averaged over the 10 random runs.

		Predicted	
		Normal	Defective
**True**	**Normal**	94.45%	5.55%
	**Defective**	0.00%	100.00%

**Table 4:** Model performance summary based on the pile type-wise cross validation.

Pile Type	# of Piles	# of Defect Piles	TPR (%)	FPR (%)
I	40	0	-	0.20
II	418	16	93.75	4.78
III	345	31	96.77	5.51
IV	120	16	87.50	5.83
Total	923	63		
